# Genetic link between primary sclerosing cholangitis and thyroid dysfunction: a bidirectional two-sample Mendelian randomization study

**DOI:** 10.3389/fimmu.2023.1276459

**Published:** 2023-10-19

**Authors:** Wenhui Zhang, Ren Lang

**Affiliations:** Department of Hepatobiliary Surgery, Beijing Chao-Yang Hospital Affiliated to Capital Medical University, Beijing, China

**Keywords:** primary sclerosing cholangitis, thyroid dysfunction, hyperthyroidism, hypothyroidism, Mendelian randomization, causal relationship

## Abstract

**Background:**

Observational studies have demonstrated an association between primary sclerosing cholangitis (PSC) and thyroid dysfunction (TD). However, the causal relationship between PSC and TD remains uncertain. The purpose of this study is to investigate the causal associations and specific direction between these two conditions. Gaining insight into the potential causal relationship between PSC and TD is valuable for elucidating the pathogenesis of PSC and for devising innovative approaches for the prevention and treatment of PSC and its associated complications.

**Methods:**

We conducted a bidirectional two-sample Mendelian randomization (MR) analysis to investigate the causal association between PSC and TD, such as autoimmune thyroid disease (AITD), thyroid cancer (TC), thyroid stimulating hormone (TSH), thyrotropin-releasing hormone (TRH), among others. PSC was the exposure variable, while TD was the outcome variable. To identify suitable instrumental variables (IVs), we utilized genome-wide association study (GWAS) datasets to select potential candidate single-nucleotide polymorphisms (SNPs). The primary statistical approach employed was the inverse-variance weighted (IVW) method, which was complemented by a series of sensitivity analyses to assess the robustness of the results by estimating heterogeneity and pleiotropy.

**Results:**

We found that the causal associations between genetically predicted PSC and Graves’ disease (GD), hyperthyroidism (IVW OR=1.230, 95%CI: 1.089–1.389, P=0.001; IVW OR=1.001, 95%CI: 1.000-1.002, P=0.000) were statistically significant. The reverse MR analysis indicated that genetic susceptibility to hyperthyroidism (P=0.000) and hypothyroidism (p=0.028) might be the risk of PSC. There was no statistically significant causal association observed between PSC and other TD (IVW P>0.05), with the exception of GD, hyperthyroidism, and hypothyroidism as determined through bidirectional two-sample analysis. To ensure the reliability of our findings, additional sensitivity analyses were conducted, including the leave-one-out (LOO) test, heterogeneity test, and pleiotropic test.

**Conclusion:**

In this study, we conducted an investigation into the causal association between PSC and TD. Our findings indicate that PSC significantly elevates the susceptibility to GD and hyperthyroidism from a statistical perspective. These results shed light on the etiology of PSC and have implications for the management of patients with PSC.

## Introduction

Primary sclerosing cholangitis (PSC) is characterized by autoimmune cholestatic liver disease, resulting in the development of multifocal biliary strictures through inflammation and fibrosis (1, 2). The rarity of PSC is evident, as it affects fewer than 200,000 individuals in the United States (US) and less than 5 per 10,000 inhabitants in the European Union (EU). The prevalence and annual incidence rate of PSC are estimated to be approximately 1 per 10,000 and 0.4 to 20 cases per 100,000 per year in the US and Northern Europe, respectively (3, 4). Furthermore, population-based studies have indicated a yearly rise in the incidence and prevalence of PSC, potentially attributed in part to the utilization of magnetic resonance cholangiography (MRC) (5–8). PSC is distinguished by the progressive deterioration of liver function, often accompanied by a heightened susceptibility to cholangiocarcinoma and colorectal cancer. In cases of advanced-stage PSC, liver transplantation (LT) becomes necessary. However, there is a possibility of disease recurrence following LT or surgical intervention (9). Despite the proposal of various mechanistic theories, the pathogenesis of PSC remains unclear and intricate. Consequently, comprehending the potential pathogenesis of PSC is of utmost importance in order to facilitate the development of efficacious therapeutic approaches and enhance prognosis.

It is well-established that patients with PSC exhibit distinct characteristics that are linked to a range of extrahepatic autoimmune diseases, such as rheumatoid arthritis (RA), scleroderma, inflammatory bowel disease (IBD), and autoimmune thyroid disease (AITD) (10, 11). A comparative study has examined the prevalence of TD in PSC, revealing a rate of 11%, and an incidence of 2.1 patients per 100 person-years (12). The findings of this study revealed that TD was unexpectedly prevalent among patients with PSC, surpassing the expected prevalence in the general population (12). Therefore, additional research on the association between thyroid disease and PSC is justified. Furthermore, a separate investigation involving PSC patients reported a prevalence of thyroid diseases in 8.4% of the 119 participants (13). Indeed, the relationship between PSC and AITD has been examined through descriptive and observational studies (14). In addition, there exists a significant correlation between thyroid hormones and liver function, and it is crucial to promptly diagnose and manage TD in patients with PSC to enhance the quality of their healthcare. A case report has documented the occurrence of hyperthyroidism, specifically Graves’ disease (GD), in a 19-year-old male patient with PSC and a history of ulcerative colitis. This observation suggests a shared immunogenetic predisposition between PSC and GD, potentially influenced by the presence of a human leukocyte antigen (HLA) phenotype (15). This finding demonstrates that patients with PSC are at an elevated risk for AITD, encompassing both GD and Hashimoto’s thyroiditis, also referred to as autoimmune thyroiditis (AT). Research indicates that PSC patients exhibit higher levels of serum T3, T4, and thyroid-stimulating hormone (TSH) in comparison to control groups (16). However, the establishment of a causal relationship between PSC and TD is derived from observational studies, wherein the presence of reverse causality, selection bias, and particularly unobserved confounding factors may obscure genuine causal connections. Consequently, it is crucial to investigate the correlation between PSC and TD in order to gain insights into the fundamental mechanisms of these diseases and improve their therapeutic approaches and quality of life.

Priority of the double-blind randomized controlled trial (RCT) may be compromised due to inherent disadvantages including challenging ethical approval, substantial time, manpower, and financial investments (17). In our study, Mendelian randomization (MR) was employed as a quasi- RCT design for analyzing causal effects.

MR can be conducted by utilizing single-nucleotide polymorphisms (SNPs) as instrumental variables (IVs) to establish a causal association between exposure and outcome (18, 19). This approach is widely acknowledged in observational studies, relying on the assumptions of IVs (20, 21). MR leverages genotypes that remain unaltered by confounding factors, thus serving as a corrective measure for the bias introduced by the presence of confounding factors in observational studies (22). The natural advantage of MR is determined by the principles of Mendel’s law of inheritance. The process of meiosis entails the random distribution of homologous chromosomes and the subsequent combination of gametes, leading to the random assortment of genetic variations in the absence of external influences (23, 24). In contrast to alternative research methodologies, MR demonstrates a reduced level of measurement error concerning genetic variation and its subsequent effects. Furthermore, the utilization of genome-wide association studies (GWAS) in the context of MR is relatively uncomplicated, particularly in bidirectional two-sample MR (25). In this scenario, MR is based on the premise that if a causal connection exists between PSC and TD, SNPs linked to the exposures will also demonstrate an association with the outcomes, thereby establishing a causal link between PSC and TD through the mediation of exposures. The IVs must adhere to the following criteria (1): demonstrate a robust correlation with the exposure factors (2), should not be linked to any other potential confounding factors, and (3) should not possess a direct association with the outcome (24). Therefore, we employed MR analyses utilizing valid SNPs as IVs to ascertain and evaluate the causal relationship between PSC and TD. This investigation was based on twelve extensive GWAS summary statistics, predominantly focusing on the European population’s data pertaining to PSC and TD, including AITD (GD and AT), hyperthyroidism, hypothyroidism, thyroid cancer (TC), TSH, thyrotropin-releasing hormone (TRH), thyroxine-binding globulin (TBG), thyroid hormone receptor alpha (THRα), thyroid peroxidase (TP) and thyroglobulin (TG). In addition, in order to ensure the reliability of our findings, we conducted various sensitivity analyses, encompassing the heterogeneity test, pleiotropy test, leave-one-out (LOO) test, and reverse-direction MR analyses.

## Methods

### Source of data and study design

The analysis was conducted using publicly accessible summary-level data from GWAS that specifically examined traits of interest, primarily in individuals of European ancestry, encompassing both males and females. A total of twelve datasets on PSC and TD traits were collected. The PSC dataset (n= 14890) was obtained from the International PSC Study Group (IPSCSG) as reported by Sun-Gou Ji et al., which investigated the relationship between the quantity of PSC and SNPs (26). AITD dataset includes both AT (n=212453) and GD (n=NA). Hyperthyroidism dataset (n=337159) was obtained from Neale Lab Consortium. Hypothyroidism dataset contained 463010 individuals from MRC-IEU in European population. TC dataset (n=1080) was obtained from the data reported by Aleksandra Köhler et al (27). TSH, TRH, THRα, TP and TG dataset all contained 3301 individuals in European population (28). TBG dataset was obtained from GWAS through the human blood plasma proteome (29).

**Figure 1:**
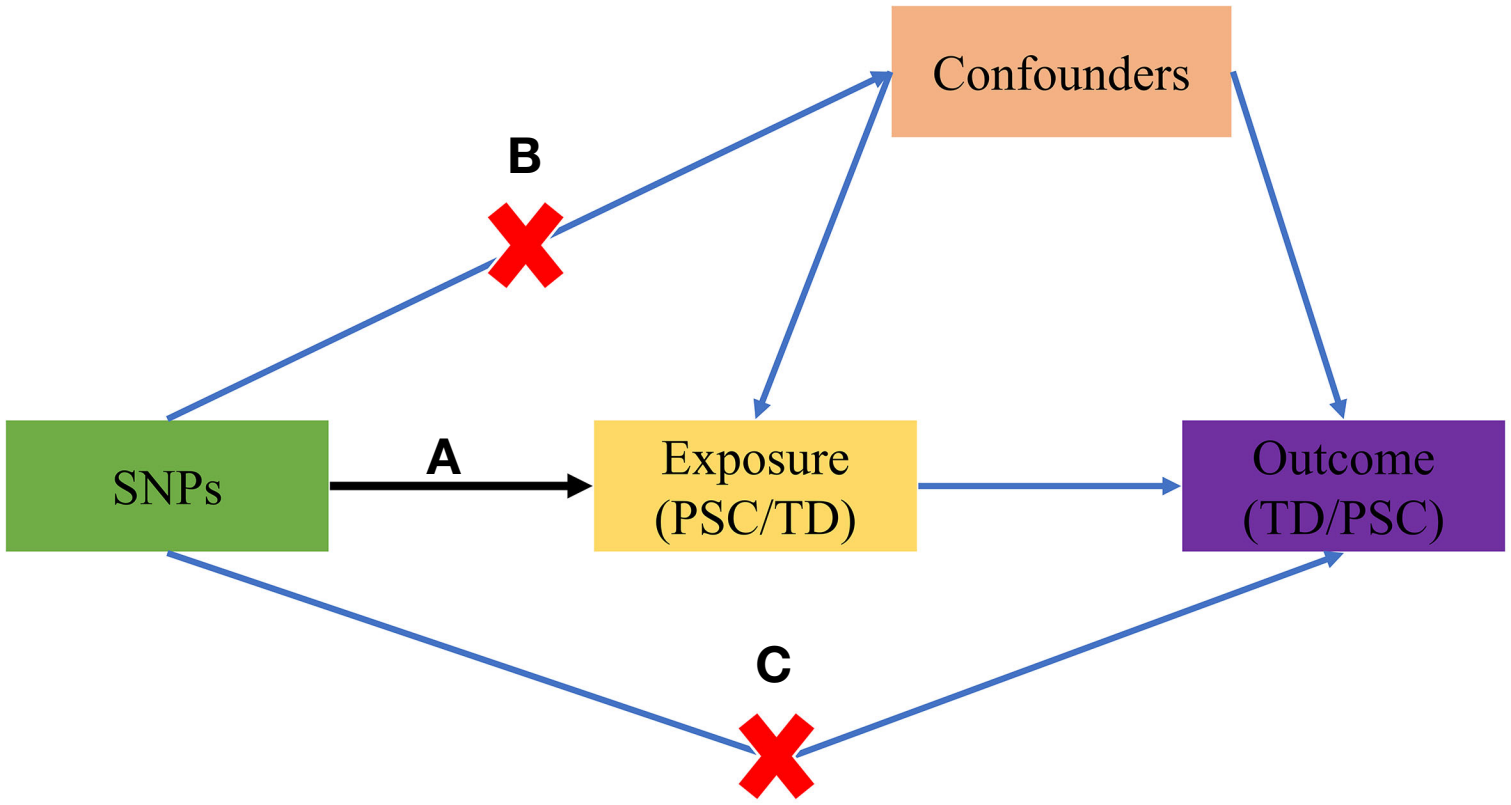
The basic principles of the MR study. **(A)** SNPs are strongly associated with exposure; **(B)** SNPs are independent of confounders; **(C)** SNPs must only affect outcome via exposure. MR, Mendelian randomization; PSC, primary sclerosing cholangitis; TD, thyroid dysfunction; SNP, single-nucleotide polymorphism.

The study design is visually presented in **Figure 1**, illustrating the schematic representation and the three core assumptions of MR. These assumptions are as follows: (A) a robust association exists between SNPs and the exposure variable (TD/PSC); (B) SNPs are not influenced by any known confounding factors; and (C) the impact of SNPs on the outcome variable (PSC/TD) is solely mediated through the exposure variable (TD/PSC).

### Selection and validation of IVs

A series of quality control procedures were implemented to identify eligible SNPs that exhibit improved statistical power in the analysis of genetic variants. Initially, IVs were utilized to examine preprocessed exposure data and determine genetic variants that met the genome-wide association threshold (P<5*10^−8^) (30, 31). In the event that the number of filtered SNPs was insufficient, it was feasible to modify the threshold to P<1*10^-5^. Following this, a manual search was conducted using PhenoScanner V2 to mitigate the potential impact of confounding factors and identify SNPs associated with the outcome (32). Thirdly, variants within a physical proximity of less than 10000 kb and with an R^2^<0.001 were excluded to reduce the presence of linkage disequilibrium (LD). We also excluded SNPs for the existence of palindromic sequences with intermediate allele frequencies. Moreover, the F-statistic was calculated to evaluate the effectiveness of individual SNPs (33). SNPs with F-statistics exceeding 10 were deemed sufficiently robust to counteract potential biases (30). SNPs with a minor allele frequency below 5% were ultimately excluded from the analysis. The present analysis did not require ethics approval as all GWAS data included in the study were publicly available and had already obtained approval from the respective ethical review boards.

### MR design and statistical analyses

The exposure and outcome datasets were harmonized in order to ensure consistent reference to the same allele (34). By selecting the SNPs as IVs, it was necessary for them to be valid. As the primary analysis, the inverse-variance weighted (IVW) method was employed, along with four supplementary methods (MR-Egger, weighted median, weighted mode, and simple mode), to investigate the potential causal effect between PSC and TD. Additionally, reverse-direction MR was conducted to evaluate the potential reverse causal association of TD on PSC.

### Sensitivity analysis

**Figure 2:**
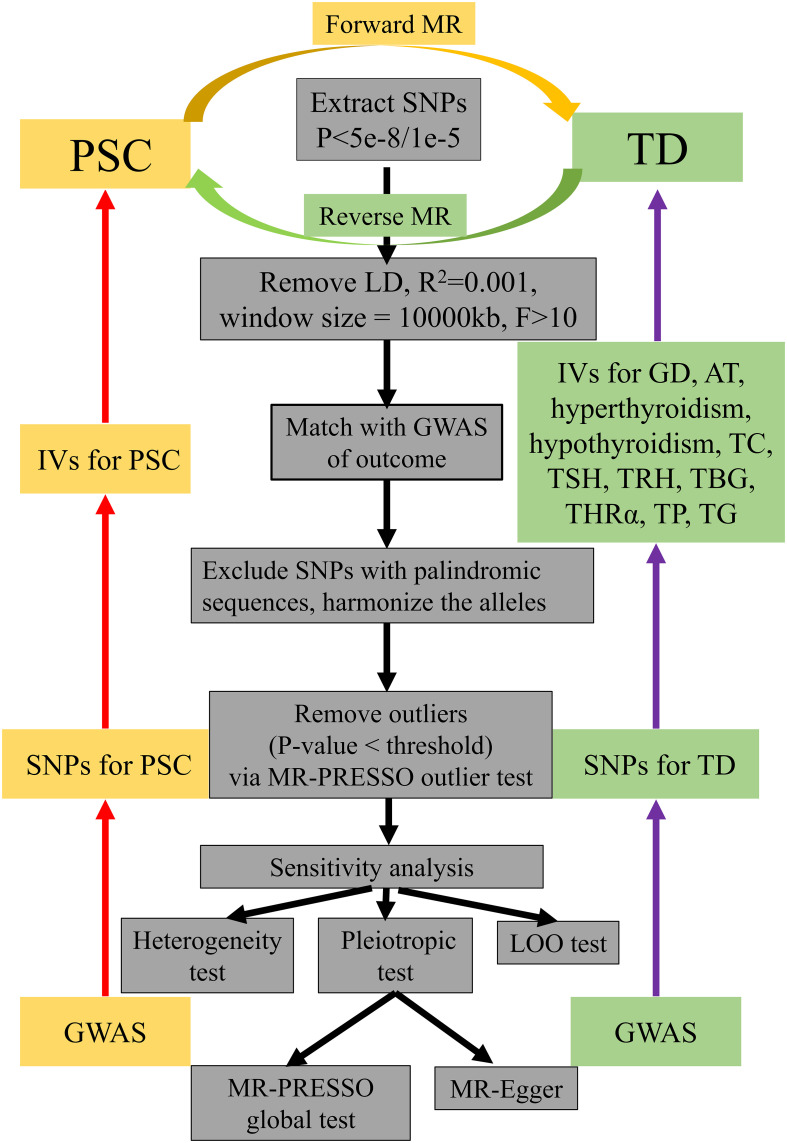
A flowchart about the screening of IVs and how the MR analysis was performed. IVs, instrumental variables; MR, Mendelian randomization; PSC, primary sclerosing cholangitis; TD, thyroid dysfunction; LD, linkage disequilibrium; GWAS, genome-wide association study; LOO test, leave-one-out test; SNP, single-nucleotide polymorphism; GD, Graves’ disease; AT, autoimmune thyroiditis; TC, thyroid cancer; TSH, thyroid stimulating hormone; TRH, thyrotropin-releasing hormone, TBG, thyroxine-binding globulin; THRα, thyroid hormone receptor alpha; TP, thyroid peroxidase; TG, thyroglobulin.

A sensitivity analysis was conducted to assess potential deviations from the model assumptions in the MR analysis. This analysis involved three tests (1): the LOO test (2), the heterogeneity test, and (3) the pleiotropic test (31, 32). The LOO test was employed to examine if individual SNPs disproportionately influenced the overall estimates. This was achieved by sequentially removing each SNP and applying IVW method to the remaining SNPs (32). Heterogeneity among the IVs was assessed using Cochrane’s Q-value (35). The results of the heterogeneity test revealed the presence of heterogeneity when the p-value was below 0.05. In order to address the potential influence of heterogeneity on the outcomes, the multiplicative random-effects model was utilized. Alternatively, the fixed effects model was employed to evaluate the causal relationship between PSC and TD. Furthermore, MR-Egger regression test and MR-PRESSO global test were utilized to assess the impact of horizontal pleiotropy and ensure that the selected IVs met the fundamental assumptions of the MR analysis (36). When the p-value is less than the predetermined significance level of 0.05, the obtained results demonstrate instability (36). Furthermore, the MR-PRESSO outlier test was used to correct SNPs by removing outliers. MR-PRESSO distortion test was employed to ascertain the presence of a statistically significant disparity in casual estimation before and after the correction of outliers. All statistical analyses were conducted using the “TwoSampleMR” (version 0.5.4) and “MRPRESSO” (Version 1.0) packages in R version 4.0.3 (R Foundation for Statistical Computing, Vienna, Austria). Statistical significance was defined as a p-value less than 0.05. The flowchart about the screening of IVs and the steps of MR analysis was shown in **Figure 2**.

## Results

In summary, the studies incorporated in this analysis were published within the timeframe of 2013 to 2021 and primarily focused on the European population, as per the specified selection criteria (**Supplementary Table S1**).

### The causal effect of PSC on TD via forward MR

**Figure 3:**
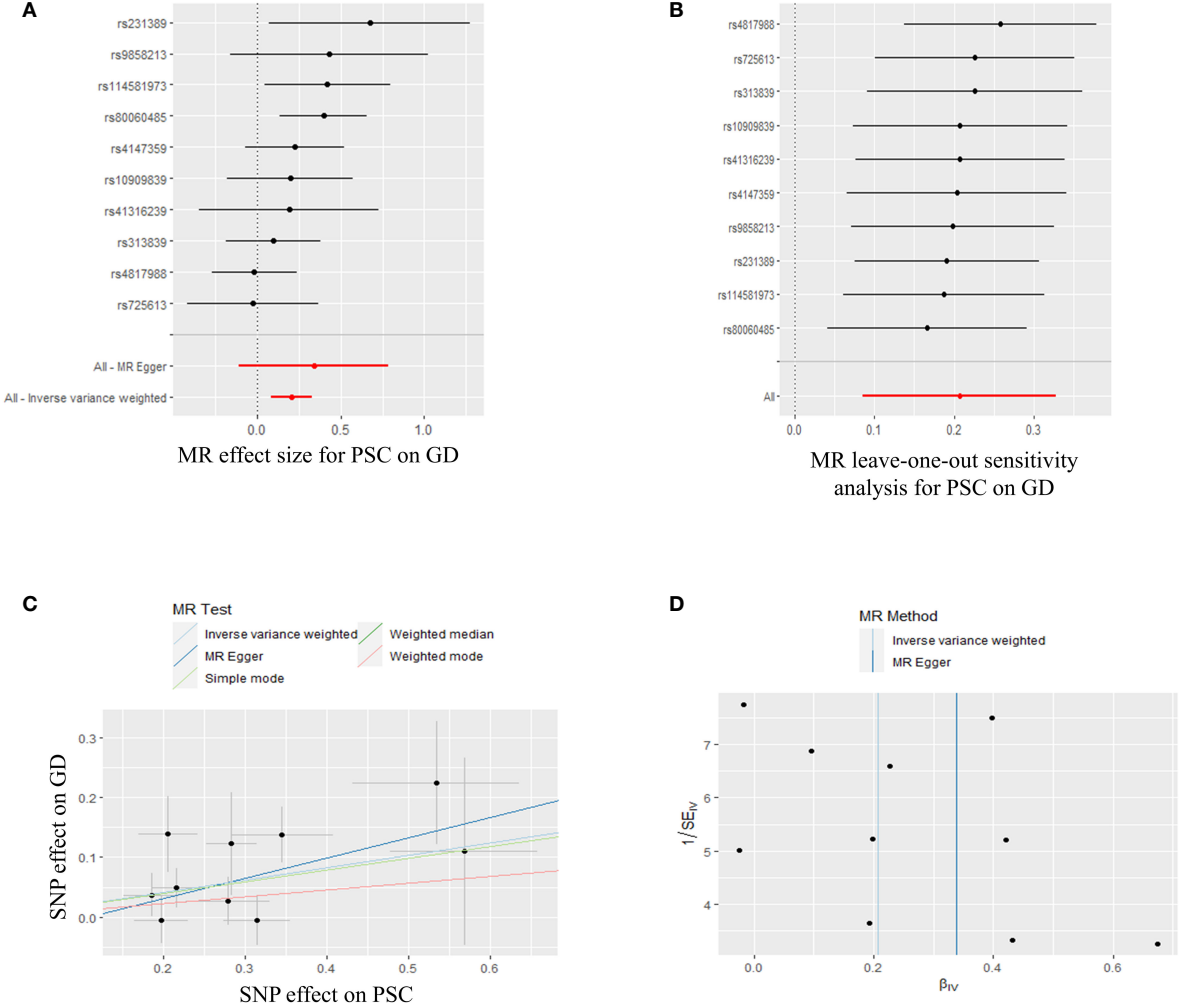
Plots of MR estimates of the causal relationship between PSC and GD. **(A)** The forest plot of SNPs associated with PSC and their risk on GD. **(B)** LOO sensitivity analysis of the association of PSC on GD. **(C)** Scatter plot of the association of PSC on GD. **(D)** Funnel plot of the association of PSC on GD. MR, Mendelian randomization; PSC, primary sclerosing cholangitis; GD, Graves’ disease; SNP, single-nucleotide polymorphism; LOO, leave-one-out.

**Figure 4:**
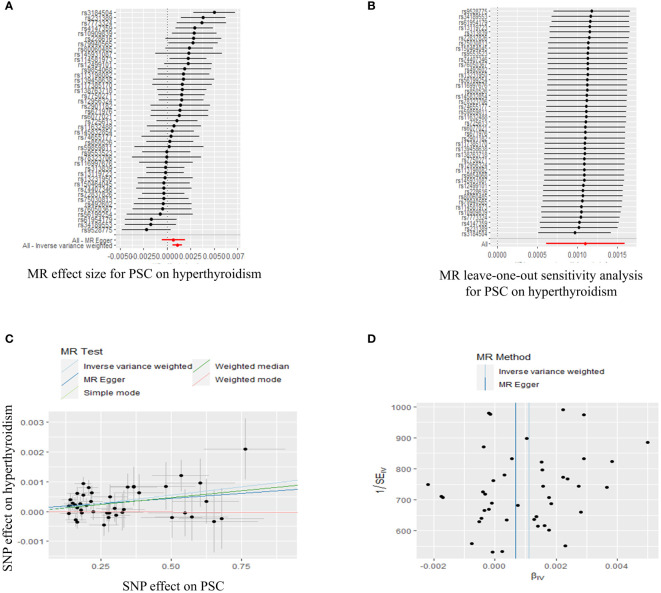
Plots of MR estimates of the causal relationship between PSC and hyperthyroidism. **(A)** The forest plot of SNPs associated with PSC and their risk on hyperthyroidism. **(B)** LOO sensitivity analysis of the association of PSC on hyperthyroidism. **(C)** Scatter plot of the association of PSC on hyperthyroidism. **(D)** Funnel plot of the association of PSC on hyperthyroidism. MR, Mendelian randomization; PSC, primary sclerosing cholangitis; SNP, single-nucleotide polymorphism; LOO, leave-one-out.

In order to conduct a thorough assessment of the connections between PSC and the likelihood of TD development, MR analyses were employed to validate associations with a statistically significant P-value of less than 0.05. The presence of genetic variations in PSC was found to be causally linked to the risk of TD. Considering the criteria for selection, specific SNPs associated with TD were retrieved for the purpose of investigating the causal impact of PSC on TD (**Supplementary Table S2**). We observed a significant causal association using the IVW method between PSC and TD (PSC and GD, IVW OR = 1.230, 95% CI 1.089-1.389, P=0.001; PSC and hyperthyroidism, IVW OR = 1.001, 95% CI 1.000-1.002, P=0.000) (**Figures 3**, **4**; **Supplementary Table S2**). Significant and consistent results between PSC and GD (P=0.017), PSC and hyperthyroidism (P=0.004) were also observed in the weighted median method (**Supplementary Table S2**). The robustness of the MR analysis findings can be further substantiated by employing the LOO method of sensitivity analyses to ascertain the impact of individual genetic variants on the overall outcomes (**Figures 3B**, **4B**). The absence of significant horizontal pleiotropy was indicated by the MR-Egger regression test and MR-PRESSO global test (PSC for GD, P=0.562; P=0.334) (**Figure 3C**; **Supplementary Table S4**). These results may suggest causal relationship between PSC and GD was stable and unbiased. On the contrary, there had a significant horizontal pleiotropy in PSC on hyperthyroidism via MR-PRESSO global test (P<0.001) while after distorting outliers using the MR- PRESSO distortion test, there was still significant difference between results before and after outlier’s correction in PSC on hyperthyroidism (P<0.001) (**Figure 4C**; **Supplementary Table S4**). This may indicate causal link between PSC and hyperthyroidism was unstable and biased. Cochran’s Q statistics test identified statistical heterogeneity using IVW (IVW PSC on GD and hyperthyroidism; Cochran’s Q=11.121, P=0.267; Cochran’s Q=62.468, P=0.023), as presented in **Figures 3D**, **4D**; **Supplementary Table S4**. Hence, IVW method used a fixed-effect model between PSC and GD, while they used a multiplicative random effect model to infer causal relationship between PSC and hyperthyroidism. MR analysis showed that there had no causal association between PSC and hypothyroidism, AT, TC, TSH, TRH, TBG, THRα, TP and TG (**Supplementary Figures S1**, **S4**-**S7**, **Supplementary Tables S2, S4**).

In summary, the findings from the PSC and GD, hyperthyroidism causally linked to MR estimation outcomes, along with the inclusion of all sensitivity analyses as qualitative control measures, collectively indicate a weak bias in the causal association.

### The causal effect of TD on PSC via reverse MR

In the context of reverse MR analysis, we conducted a study using TD as the exposure and PSC as the outcome in order to investigate the causal effects of TD on PSC. **Supplementary Table S3** presents the selected SNPs utilized as IVs to detect causality. The IVW analysis revealed a significant association between genetically predicted occurrence of two out of the eleven TD-related diseases or biomarkers and PSC, including hyperthyroidism (IVW P=0.000) and hypothyroidism (IVW P=0.028) (**Supplementary Figures S2**, **S3**, **Supplementary Table S3**). The LOO method was used to assess robustness of these results (**Supplementary Figures S2B**, **S3B**). MR-Egger regression test was executed to assess horizontal pleiotropy, and the results revealed that the presence of horizontal pleiotropy was unlikely to bias the causality of hyperthyroidism (intercept=0.056, P=0.329) (**Supplementary Figure S2C**, **Supplementary Table S4**) and hypothyroidism with PSC (intercept=0.045, P=0.155) (**Supplementary Figure S3C**, **Supplementary Table S4**). It is worth noting that there had significant difference between results before and after outlier’s correction in hyperthyroidism and hypothyroidism for PSC after distorting outliers using the MR- PRESSO distortion test (P<0.001). Heterogeneity test using IVW revealed that there had heterogeneity in the estimations of the SNPs included. Hence, we used a multiplicative random effect model to test causal association (hyperthyroidism on PSC, Cochran’s Q=80.234, P=0.000) (**Supplementary Figure S2D**, **Supplementary Table S4**). There had no obvious heterogeneity in hypothyroidism on PSC. Therefore, we used a fixed effects model to infer causal link between hypothyroidism on PSC (Cochran’s Q=21.034, P=0.072) (**Supplementary Figure S3D**, **Supplementary Table S4**).

Moreover, the summary data accessed from IVW analysis implied no evidence to measure causality between GD (P=0.945), AT (P=0.757), TC (P=0.396), TSH (P=0.841), TRH (P=0.770), THRα (P=0.901), TP (P=0.111), TG (P=0.469) and PSC (**Supplementary Figures S4**-**S7**, **Supplementary Tables S3, S4**). It is worth mentioning that the number of SNPs for IVs was too small based on our screening criteria, so we did not determine the causal relationship between TBG and PSC.

Forest plot, the results of the LOO sensitivity analysis, scatter plot, and the funnel plot of the link between TD and PSC are shown in **Supplementary Figures S4**-**S7**, respectively, where similar results can be observed.

## Discussion

As is known to all, this is the first study to use two-sample MR analysis and large-scale GWAS datasets to conclude a causal relationship between PSC and TD. Our study found statistical evidence that genetically predicted PSC had a positive causal effect on GD and hyperthyroidism risk, suggesting that PSC per se may play a causal role in the pathogenesis of TD. However, there was no causal effect between PSC and AT, hypothyroidism, TC, TSH, TRH, TBG, THRα, TP, TG. Moreover, the reverse MR analysis suggested that genetic susceptibility to hyperthyroidism and hypothyroidism might affect the risk of PSC. A series of sensitive analyses supported the findings mentioned above.

In terms of the causal relationship between PSC and GD, our analysis revealed that PSC may have a potential cause-and-effect influence on GD, as indicated by MR analyses. However, the reverse-direction MR analyses did not yield any significant findings. From this review, we can infer that the genome-wide significant risk loci (chromosome: 2q33, candidate risk genes: CD28 and CTLA4, lead SNP: rs7426056) are shared between PSC and GD, excluding the HLA complex on chromosome 6 (9). Consistent with our findings, an engaging case have shown that there was combined pathogenesis rather than a coincidence in a PSC patient with GD (15). As expected, our results are consistent with previous retrospective studies (12). Prevalence of TD in patients with PSC was 11% (95% CI 0.060-0.200). Of these patients, five (6%) had hypothyroidism and four (5%) had hyperthyroidism (12).

The bidirectional two-sample MR design was employed to establish a causal relationship between PSC and both hyperthyroidism and hypothyroidism. Our results are analogous to a case report (37). M Zeniya also showed that hypothyroidism was observed in both autoimmune hepatitis and primary biliary cirrhosis (PSC), at 12% and 5.8%, respectively in Japan. The occurrence of PSC and hyperthyroidism or hypothyroidism might be caused by genetic factors (38, 39). Similarly, PBC, an autoimmune liver disease (AILD) similar to PSC, was also found to be causally related to AITD, TSH, hypothyroidism and TC by MR analysis (40). It is worth noting that the causal effect is weak between PSC and hyperthyroidism, as the OR is close to 1 (IVW OR=1.001), even though it is statistically significant. This is why it is rare in the publications and has only been reported in case reports so far.

Multiple hypotheses have been proposed regarding the causal relationship between hypothyroidism and PSC. One such hypothesis suggests that the shared occurrence of the HLA phenotype is frequently observed in individuals with both PSC and hypothyroidism (chromosome:12q24, candidate risk genes: SH2B3 and ATXN2, lead SNP: rs3184504) and the presence of several autoantibodies suggest a common immunogenetic predisposition. On the other hand, we investigated the presence of missense mutations or loss of immunostaining for 7H6, a tight junction (TJ) protein specific to bile canaliculi in hepatocytes, in patients with PSC. This protein is closely linked to paracellular permeability and bile duct injury. The clinical features of patients with TJ mutations included hypothyroxinemia (41, 42). In fact, there are few studies on PSC and hyperthyroidism/hypothyroidism in view of weak causal association. In conclusion, given the established causal relationship between PSC and hyperthyroidism/hypothyroidism, our findings make a valuable contribution to the ongoing investigation of the pathogenesis of PSC.

In our analysis using MR, we observed a significant and consistent causal relationship between PSC and TD (GD and hypothyroidism) through the use of the IVW method as the primary approach. However, the results obtained from alternative methods such as MR Egger, weighted median, simple mode, and weighted mode showed little significance. Moreover, OR values are very close to 1 with weak causal effect, although the relationship between PSC and hyperthyroidism indicates the statistical significance. This lack of significance may be attributed to the low statistical power of these methods or the potential underestimation of the causal effect due to inflated type I error when all genetic variants are assumed to be valid (43–45). With the exception of IVW and weighted median, all methods indicate no significant association between PSC and hyperthyroidism. Moreover, the use of methods to examine the causal direction of PSC on other TD did not yield statistically significant results (P>0.05).

Our results have the potential to enhance the understanding of the diagnosis and management of patients with PSC and TD. Specifically, our findings provide insights into the occurrence of TD in PSC patients, offer guidance for the treatment of TD in PSC patients, and contribute to the improvement of the quality of life for individuals with PSC. The data from our study demonstrated a causal link between PSC and TD (GD and hyperthyroidism). Consistent monitoring of thyroid-related hormones and prompt diagnosis and treatment of TD can provide valuable insights for the management of PSC and lifestyle interventions. It would be meaningful for clinicians to monitor the diagnostic markers of TD in patients diagnosed with PSC, particularly those who have risk factors associated with TD. Additionally, it is crucial to exercise caution when administering thyroid hormone-related drugs to TD patients in order to safeguard liver function and AILD. Furthermore, future research should focus on establishing the relationship between metabolites and PSC, with the aim of identifying novel and effective biomarkers that can aid in the exploration of the potential pathogenesis of PSC in TD (46). However, the results need further validation *in vivo*.

Our study is subject to several limitations. First, the use of MR analysis does not allow for the complete elimination of potential unmeasured confounding factors. Consequently, the presence of unknown confounders may compromise the validity of our IVs assumptions, thereby introducing bias into our results. Next, the participants in our study predominantly consisted of individuals from the European population, as evidenced by the GWAS data. Our conclusions in MR analysis may not apply to other populations. Moreover, there may be an overlap in the population included in both the exposure and outcome of our analysis, which could potentially affect the reliability of the results. Therefore, further investigations should be conducted on a more diverse population to ensure the generalizability of our findings. Furthermore, the IVW OR obtained from MR analysis of PSC in causal relation to hyperthyroidism was found to be 1.001. This value, being in close proximity to 1, suggest a weak causal effect. This implies that PSC is less likely to be associated with hyperthyroidism potentially due to smaller populations and lack of uniformity in diagnostic criteria, etc. Finally, it is widely acknowledged that the incidence of PSC may vary based on sex. Therefore, future studies should carefully consider the influence of sex on causality when examining this relationship.

In summary, the results of our study demonstrate that PSC can lead to TD, particularly in the forms of GD and hyperthyroidism. The identification of a potential causal association between PSC and TD offers a new avenue for investigating the origins and progression of PSC.

## Data availability statement

The original contributions presented in the study are included in the article/**Supplementary Material**. Further inquiries can be directed to the corresponding author.

## Ethics statement

Ethical review and approval was not required for the study on human participants in accordance with the local legislation and institutional requirements. Since this MR study is based on publicly available GWAS summary statistics, written informed consent for participation was not required for this study in accordance with the national legislation and the institutional requirements.

## Author contributions

WZ: Writing – review & editing, Conceptualization, Data curation, Formal Analysis, Investigation, Software, Supervision, Validation, Visualization, Writing – original draft. RL: Funding acquisition, Methodology, Resources, Supervision, Validation, Writing – review & editing.

## Supplementary material

Supplementary Figure 1 Plots of MR estimates of the causal relationship between PSC and hypothyroidism. **(A)** The forest plot of SNPs associated with PSC and their risk on hypothyroidism. **(B)** LOO sensitivity analysis of the association of PSC on hypothyroidism. **(C)** Scatter plot of the association of PSC on hypothyroidism. **(D)** Funnel plot of the association of PSC on hypothyroidism. MR, Mendelian randomization; PSC, primary sclerosing cholangitis; SNP, single-nucleotide polymorphism; LOO, leave-one-out.

Supplementary Figure 2 Plots of MR estimates of the causal relationship between hyperthyroidism and PSC. **(A)** The forest plot of SNPs associated with hyperthyroidism and their risk on PSC. **(B)** LOO sensitivity analysis of the association of hyperthyroidism on PSC. **(C)** Scatter plot of the association of hyperthyroidism on PSC. **(D)** Funnel plot of the association of hyperthyroidism on PSC. MR, Mendelian randomization; PSC, primary sclerosing cholangitis; SNP, single-nucleotide polymorphism; LOO, leave-one-out.

Supplementary Figure 3 Plots of MR estimates of the causal relationship between hypothyroidism and PSC. **(A)** The forest plot of SNPs associated with hypothyroidism and their risk on PSC. **(B)** LOO sensitivity analysis of the association of hypothyroidism on PSC. **(C)** Scatter plot of the association of hypothyroidism on PSC. **(D)** Funnel plot of the association of hypothyroidism on PSC. MR, Mendelian randomization; PSC, primary sclerosing cholangitis; SNP, single-nucleotide polymorphism; LOO, leave-one-out.

Supplementary Figure 4 Forest plot of the association of PSC on AT **(A)**, TC **(B)**, TSH **(C)**, TRH **(D)**, TBG **(E)**, THRα **(F)**, TP**(G)**, TG (H). Forest plot of the association of GD **(I)**, AT **(J)**, TC **(K)**, TSH **(L)**, TRH **(M)**, THRα **(N)**, TP **(O)**, TG **(P)** on PSC. PSC, primary sclerosing cholangitis; AT, autoimmune thyroiditis; TC, thyroid cancer; TSH, thyroid stimulating hormone; TRH, thyrotropin-releasing hormone, TBG, thyroxine-binding globulin; THRα, thyroid hormone receptor alpha; TP, thyroid peroxidase; TG, thyroglobulin.

Supplementary Figure 5 LOO sensitivity analysis of the association of PSC on AT **(A)**, TC **(B)**, TSH **(C)**, TRH **(D)**, TBG **(E)**, THRα **(F)**, TP**(G)**, TG **(H)**. LOO sensitivity analysis of the association of GD **(I)**, AT **(J)**, TC **(K)**, TSH **(L)**, TRH **(M)**, THRα **(N)**, TP **(O)**, TG **(P)** on PSC. PSC, primary sclerosing cholangitis; AT, autoimmune thyroiditis; TC, thyroid cancer; TSH, thyroid stimulating hormone; TRH, thyrotropin-releasing hormone, TBG, thyroxine-binding globulin; THRα, thyroid hormone receptor alpha; TP, thyroid peroxidase; TG, thyroglobulin.

Supplementary Figure 6 Scatter plot of the association of PSC on AT **(A)**, TC **(B)**, TSH **(C)**, TRH **(D)**, TBG **(E)**, THRα **(F)**, TP**(G)**, TG **(H)**. Scatter plot of the association of GD **(I)**, AT **(J)**, TC **(K)**, TSH **(L)**, TRH **(M)**, THRα **(N)**, TP **(O)**, TG **(P)** on PSC. PSC, primary sclerosing cholangitis; AT, autoimmune thyroiditis; TC, thyroid cancer; TSH, thyroid stimulating hormone; TRH, thyrotropin-releasing hormone, TBG, thyroxine-binding globulin; THRα, thyroid hormone receptor alpha; TP, thyroid peroxidase; TG, thyroglobulin.

Supplementary Figure 7 Funnel plot of the association of PSC on AT **(A)**, TC **(B)**, TSH **(C)**, TRH **(D)**, TBG **(E)**, THRα **(F)**, TP**(G)**, TG **(H)**. Funnel plot of the association of GD **(I)**, AT **(J)**, TC **(K)**, TSH **(L)**, TRH **(M)**, THRα **(N)**, TP **(O)**, TG **(P)** on PSC. PSC, primary sclerosing cholangitis; AT, autoimmune thyroiditis; TC, thyroid cancer; TSH, thyroid stimulating hormone; TRH, thyrotropin-releasing hormone, TBG, thyroxine-binding globulin; THRα, thyroid hormone receptor alpha; TP, thyroid peroxidase; TG, thyroglobulin.
