# Supplementary material for: Genetic link between primary sclerosing cholangitis and thyroid dysfunction: a bidirectional two-sample Mendelian randomization study
Source: Front Immunol. 2023 Oct 19;14:1276459. doi: 10.3389/fimmu.2023.1276459 (PMC10622799; doi:10.3389/fimmu.2023.1276459)
Supplement: Supplementary file 8 [file Table_1.docx]

Table S1. Baseline characteristics of PSC and TD dataset in the present study.

| Trait | Year | Author | Population | Sample Size | n case | n control | n SNP |
| --- | --- | --- | --- | --- | --- | --- | --- |
| PSC | 2017 | Ji | Mixed | 14890 | 2871 | 12019 | 7891603 |
| AITD |  |  |  |  |  |  |  |
| GD | 2019 | Ishigaki K | East Asian | 212453 | 2176 | 210277 | 8885805 |
| AT | 2021 | NA | European |  | 244 | 187684 | 16380358 |
| Hyperthyroidism | 2017 | Neale | European | 337159 | 2547 | 334612 | 10894596 |
| Hypothyroidism | 2018 | Ben Elsworth | European | 463010 | 9674 | 453336 | 9851867 |
| TC | 2013 | Kohler A | European | 1080 | 649 | 431 | 572028 |
| TSH | 2018 | Sun BB | European | 3301 |  |  | 10534735 |
| TRH | 2018 | Sun BB | European | 3301 |  |  | 10534735 |
| TBG | 2019 | Suhre K | European |  |  |  | 501428 |
| THRα | 2018 | Sun BB | European | 3301 |  |  | 10534735 |
| TP | 2018 | Sun BB | European | 3301 |  |  | 10534735 |
| TG | 2018 | Sun BB | European | 3301 |  |  | 10534735 |

PSC, primary sclerosing cholangitis; TD, thyroid dysfunction; SNP, single-nucleotide polymorphism; GD, Graves' disease; AT, autoimmune thyroiditis; TC, thyroid cancer; TSH, thyroid stimulating hormone; TRH, thyrotropin-releasing hormone, TBG, thyroxine-binding globulin; THRα, thyroid hormone receptor alpha; TP, thyroid peroxidase; TG, thyroglobulin.
