# Supplementary material for: Genetic link between primary sclerosing cholangitis and thyroid dysfunction: a bidirectional two-sample Mendelian randomization study
Source: Front Immunol. 2023 Oct 19;14:1276459. doi: 10.3389/fimmu.2023.1276459 (PMC10622799; doi:10.3389/fimmu.2023.1276459)
Supplement: Supplementary file 9 [file Table_2.docx]

Table S2. MR results of PSC on TD

| Exposure vs outcome | Methods | SNPs | OR | 95%CI | *P* |
| --- | --- | --- | --- | --- | --- |
| PSC vs GD | MR Egger | 16 | 1.403 | 0.898-2.192 | 0.175 |
|  | Weighted median | 16 | 1.217 | 1.036-1.429 | **0.017** |
|  | Inverse variance weighted | **16** | **1.230** | **1.089-1.389** | **0.001** |
|  | Simple mode | 16 | 1.216 | 0.914-1.618 | 0.212 |
|  | Weighted mode | 16 | 1.120 | 0.893-1.406 | 0.352 |
| PSC vs AT | MR Egger | 15 | 0.604 | 0.280-1.305 | 0.226 |
|  | Weighted median | 15 | 0.975 | 0.652-1.460 | 0.904 |
|  | Inverse variance weighted | 15 | 0.977 | 0.690-1.383 | 0.896 |
|  | Simple mode | 15 | 0.980 | 0.532-1.807 | 0.950 |
|  | Weighted mode | 15 | 0.995 | 0.532-1.860 | 0.988 |
| PSC vs hyperthyroidism | MR Egger | 47 | 1.001 | 0.999-1.002 | 0.289 |
|  | Weighted median | 47 | 1.001 | 1.000-1.002 | **0.004** |
|  | Inverse variance weighted | **47** | **1.001** | **1.001-1.002** | **0.000** |
|  | Simple mode | 47 | 1.000 | 0.998-1.002 | 0.963 |
|  | Weighted mode | 47 | 1.000 | 0.999-1.001 | 0.959 |
| PSC vs hypothyroidism | MR Egger | 12 | 0.996 | 0.990-1.003 | 0.313 |
|  | Weighted median | 12 | 1.000 | 0.999-1.002 | 0.781 |
|  | Inverse variance weighted | 12 | 1.001 | 0.999-1.002 | 0.368 |
|  | Simple mode | 12 | 1.001 | 0.999-1.004 | 0.432 |
|  | Weighted mode | 12 | 0.999 | 0.998-1.001 | 0.599 |
| PSC vs TC | MR Egger | 7 | 5.576 | 0.411-75.720 | 0.253 |
|  | Weighted median | 7 | 1.308 | 0.772-2.216 | 0.318 |
|  | Inverse variance weighted | 7 | 1.288 | 0.862-1.925 | 0.216 |
|  | Simple mode | 7 | 1.383 | 0.642-2.977 | 0.439 |
|  | Weighted mode | 7 | 1.371 | 0.699-0.690 | 0.394 |
| PSC vs TSH | MR Egger | 18 | 1.002 | 0.957-1.050 | 0.923 |
|  | Weighted median | 18 | 0.995 | 0.952-1.040 | 0.821 |
|  | Inverse variance weighted | 18 | 1.006 | 0.973-1.040 | 0.746 |
|  | Simple mode | 18 | 0.968 | 0.888-1.055 | 0.469 |
|  | Weighted mode | 18 | 1.004 | 0.963-1.048 | 0.848 |
| PSC vs TRH | MR Egger | 18 | 0.956 | 0.907-1.007 | 0.111 |
|  | Weighted median | 18 | 0.960 | 0.921-1.000 | 0.052 |
|  | Inverse variance weighted | 18 | 0.986 | 0.948-1.026 | 0.499 |
|  | Simple mode | 18 | 0.950 | 0.864-1.044 | 0.302 |
|  | Weighted mode | 18 | 0.958 | 0.918-0.999 | 0.062 |
| PSC vs TBG | MR Egger | 7 | 1.161 | 0.894-1.506 | 0.313 |
|  | Weighted median | 7 | 1.064 | 0.880-1.286 | 0.523 |
|  | Inverse variance weighted | 7 | 0.917 | 0.779-1.080 | 0.298 |
|  | Simple mode | 7 | 0.771 | 0.507-1.173 | 0.271 |
|  | Weighted mode | 7 | 1.078 | 0.893-1.301 | 0.465 |
| PSC vs THRα | MR Egger | 18 | 0.981 | 0.936-1.028 | 0.423 |
|  | Weighted median | 18 | 0.960 | 0.913-1.009 | 0.107 |
|  | Inverse variance weighted | 18 | 1.000 | 0.967-1.033 | 0.985 |
|  | Simple mode | 18 | 0.960 | 0.869-1.062 | 0.440 |
|  | Weighted mode | 18 | 0.957 | 0.902-1.015 | 0.161 |
| PSC vs TP | MR Egger | 18 | 0.974 | 0.928-1.023 | 0.309 |
|  | Weighted median | 18 | 0.980 | 0.939-1.203 | 0.361 |
|  | Inverse variance weighted | 18 | 0.984 | 0.951-1.018 | 0.358 |
|  | Simple mode | 18 | 0.972 | 0.900-1.049 | 0.472 |
|  | Weighted mode | 18 | 0.979 | 0.940-1.019 | 0.306 |
| PSC vs TG | MR Egger | 18 | 0.989 | 0.939-1.043 | 0.697 |
|  | Weighted median | 18 | 0.990 | 0.945-1.037 | 0.667 |
|  | Inverse variance weighted | 18 | 0.985 | 0.950-1.022 | 0.424 |
|  | Simple mode | 18 | 1.024 | 0.934-1.122 | 0.620 |
|  | Weighted mode | 18 | 0.981 | 0.939-1.024 | 0.389 |

MR, Mendelian randomization; PSC, primary sclerosing cholangitis; TD, thyroid dysfunction; SNP, single-nucleotide polymorphism; OR, odds ratio; CI, confidence interval; GD, Graves' disease; AT, autoimmune thyroiditis; TC, thyroid cancer; TSH, thyroid stimulating hormone; TRH, thyrotropin-releasing hormone, TBG, thyroxine-binding globulin; THRα, thyroid hormone receptor alpha; TP, thyroid peroxidase; TG, thyroglobulin.
