# Supplementary material for: Genetic link between primary sclerosing cholangitis and thyroid dysfunction: a bidirectional two-sample Mendelian randomization study
Source: Front Immunol. 2023 Oct 19;14:1276459. doi: 10.3389/fimmu.2023.1276459 (PMC10622799; doi:10.3389/fimmu.2023.1276459)
Supplement: Supplementary file 10 [file Table_3.docx]

Table S3. MR results of TD on PSC

| Exposure vs outcome | Methods | SNPs | OR | 95%CI | *P* |
| --- | --- | --- | --- | --- | --- |
| GD vs PSC | MR Egger | 6 | 0.792 | 0.162-3.862 | 0.787 |
|  | Weighted median | 6 | 0.995 | 0.826-1.199 | 0.957 |
|  | Inverse variance weighted | 6 | 1.010 | 0.754-1.353 | 0.945 |
|  | Simple mode | 6 | 0.887 | 0.696-1.131 | 0.379 |
|  | Weighted mode | 6 | 0.911 | 0.736-1.129 | 0.434 |
| AT vs PSC | MR Egger | 7 | 0.963 | 0.905-1.025 | 0.292 |
|  | Weighted median | 7 | 1.005 | 0.961-1.051 | 0.829 |
|  | Inverse variance weighted | 7 | 0.995 | 0.960-1.030 | 0.757 |
|  | Simple mode | 7 | 1.004 | 0.940-1.071 | 0.917 |
|  | Weighted mode | 7 | 1.005 | 0.949-1.065 | 0.870 |
| Hyperthyroidism vs PSC | MR Egger | 21 | 8.561E+02 | 0.000-1.669E+36 | 0.865 |
|  | Weighted median | 21 | 1.933E+09 | 0.453- 8.255E+18 | 0.059 |
|  | Inverse variance weighted | **21** | 1.100E+19 | 2.472E+08-4.895E+29 | **0.000** |
|  | Simple mode | 21 | 7.239E+02 | 0.000-2.652E+17 | 0.704 |
|  | Weighted mode | 21 | 2.745E+04 | 0.000- 4.879E+20 | 0.598 |
| Hypothyroidism vs PSC | MR Egger | 22 | 2.680E+16 | 3.874-1.8537E+32 | 0.065 |
|  | Weighted median | 22 | 6.286E+04 | 0.675- 5.851E+09 | 0.058 |
|  | Inverse variance weighted | **22** | 3.267E+04 | 3.150-3.388E+08 | **0.028** |
|  | Simple mode | 22 | 9.547E+03 | 0.000-1.903E+13 | 0.417 |
|  | Weighted mode | 22 | 2.273E+04 | 0.000-1.164E+14 | 0.395 |
| TC vs PSC | MR Egger | 266 | 1.003 | 0.997-1.009 | 0.385 |
|  | Weighted median | 266 | 1.004 | 0.998-1.009 | 0.212 |
|  | Inverse variance weighted | 266 | 0.998 | 0.995-1.002 | 0.396 |
|  | Simple mode | 266 | 1.001 | 0.990-1.012 | 0.888 |
|  | Weighted mode | 266 | 1.002 | 0.996-1.009 | 0.471 |
| TSH vs PSC | MR Egger | 15 | 1.215 | 0.713-2.071 | 0.486 |
|  | Weighted median | 15 | 0.934 | 0.768-1.137 | 0.497 |
|  | Inverse variance weighted | 15 | 1.018 | 0.854-1.213 | 0.841 |
|  | Simple mode | 15 | 0.932 | 0.678-1.281 | 0.671 |
|  | Weighted mode | 15 | 0.924 | 0.685-1.245 | 0.610 |
| TRH vs PSC | MR Egger | 15 | 0.857 | 0.596-1.234 | 0.422 |
|  | Weighted median | 15 | 0.995 | 0.826-1.198 | 0.957 |
|  | Inverse variance weighted | 15 | 1.021 | 0.886-1.177 | 0.770 |
|  | Simple mode | 15 | 1.120 | 0.825-1.520 | 0.480 |
|  | Weighted mode | 15 | 1.073 | 0.774-1.488 | 0.680 |
| TBG vs PSC | MR Egger | ——————————————— | | | |
|  | Weighted median |  |  |  |  |
|  | Inverse variance weighted |  |  |  |  |
|  | Simple mode |  |  |  |  |
|  | Weighted mode |  |  |  |  |
|  | MR Egger | 11 | 1.071 | 0.722-1.589 | 0.740 |
|  | Weighted median | 11 | 0.999 | 0.808-1.236 | 0.995 |
| THRα vs PSC | Inverse variance weighted | 11 | 1.010 | 0.858-1.191 | 0.901 |
|  | Simple mode | 11 | 1.005 | 0.739-1.368 | 0.975 |
|  | Weighted mode | 11 | 0.979 | 0.712-1.347 | 0.899 |
|  | MR Egger | 14 | 0.860 | 0.579-1.277 | 0.469 |
|  | Weighted median | 14 | 0.934 | 0.769-1.134 | 0.491 |
| TP vs PSC | Inverse variance weighted | 14 | 0.886 | 0.763-1.028 | 0.111 |
|  | Simple mode | 14 | 0.731 | 0.509-1.052 | 0.116 |
|  | Weighted mode | 14 | 1.100 | 0.780-1.551 | 0.597 |
| TG vs PSC | MR Egger | 10 | 0.732 | 0.525-1.020 | 0.102 |
|  | Weighted median | 10 | 0.871 | 0.672-1.128 | 0.295 |
|  | Inverse variance weighted | 10 | 0.927 | 0.756-1.138 | 0.469 |
|  | Simple mode | 10 | 0.800 | 0.557-1.149 | 0.258 |
|  | Weighted mode | 10 | 0.808 | 0.564-1.158 | 0.276 |

MR, Mendelian randomization; PSC, primary sclerosing cholangitis; TD, thyroid dysfunction; SNP, single-nucleotide polymorphism; OR, odds ratio; CI, confidence interval; GD, Graves' disease; AT, autoimmune thyroiditis; TC, thyroid cancer; TSH, thyroid stimulating hormone; TRH, thyrotropin-releasing hormone, TBG, thyroxine-binding globulin; THRα, thyroid hormone receptor alpha; TP, thyroid peroxidase; TG, thyroglobulin.
