# Supplementary material for: Genetic link between primary sclerosing cholangitis and thyroid dysfunction: a bidirectional two-sample Mendelian randomization study
Source: Front Immunol. 2023 Oct 19;14:1276459. doi: 10.3389/fimmu.2023.1276459 (PMC10622799; doi:10.3389/fimmu.2023.1276459)
Supplement: Supplementary file 11 [file Table_4.docx]

Table S4. Pleiotropy and heterogeneity test between PSC and TD

| Exposure | Outcome | Heterogeneity | | |  |  |  |  | | | | Model^*^ |
| --- | --- | --- | --- | --- | --- | --- | --- | --- | --- | --- | --- | --- |
|  |  |  |  |  | MR-PRESSO | | | | Pleiotropy |  | |  |
|  |  | IVW | | | Outlier-corrected | | Distortion Test | Global test |  | MR-Egger | |  |
|  |  | *Q* | *df* | *P* | *No.* | *P* | *P* | *P* | *Intercept* | *Se* | *P* |  |
| PSC | GD | 11.121 | 9 | 0.267 | 0 | NA | NA | 0.334 | -0.037 | 0.060 | 0.562 | 1 |
|  | AT | 24.254 | 12 | 0.062 | 2 | 0.378 | 0.755 | 0.002 | 0.150 | 0.110 | 0.201 | 1 |
|  | Hyperthyroidism | 62.468 | 42 | 0.023 | 3 | 0.000 | <0.001 | **<0.001** | 0.000 | 0.000 | 0.471 | 2 |
|  | Hypothyroidism | 10.265 | 7 | 0.174 | 4 | 0.135 | <0.001 | <0.001 | 0.001 | 0.001 | 0.246 | 1 |
|  | TC | 3.513 | 6 | 0.742 | 0 | NA | NA | 0.768 | -0.243 | 0.218 | 0.316 | 1 |
|  | TSH | 14.471 | 17 | 0.634 | 0 | NA | NA | 0.671 | 0.002 | 0.012 | 0.854 | 1 |
|  | TRH | 23.911 | 17 | 0.122 | 0 | NA | NA | 0.133 | 0.022 | 0.013 | 0.113 | 1 |
|  | TBG | 7.548 | 6 | 0.273 | 0 | NA | NA | 0.253 | -0.067 | 0.031 | 0.085 | 1 |
|  | THRα | 13.915 | 17 | 0.673 | 0 | NA | NA | 0.459 | 0.013 | 0.012 | 0.267 | 1 |
|  | TP | 17.756 | 17 | 0.404 | 0 | NA | NA | 0.496 | 0.007 | 0.012 | 0.569 | 1 |
|  | TG | 20.357 | 17 | 0.256 | 0 | NA | NA | 0.295 | -0.003 | 0.013 | 0.827 | 1 |
| GD | PSC | 22.390 | 5 | 0.000 | 1 | 0.933 | 0.043 | **0.003** | 0.057 | 0.184 | 0.774 | 2 |
| AT |  | 3.729 | 6 | 0.713 | 0 | NA | NA | 0.779 | 0.059 | 0.048 | 0.279 | 1 |
| Hyperthyroidism |  | 80.234 | 20 | 0.000 | 9 | 0.002 | <0.001 | **<0.001** | 0.056 | 0.056 | 0.329 | 2 |
| Hypothyroidism |  | 21.034 | 13 | 0.072 | 7 | 0.034 | <0.001 | **<0.001** | -0.069 | 0.045 | 0.155 | 1 |
| TC |  | 271.490 | 265 | 0.379 | 0 | NA | NA | 0.387 | -0.006 | 0.004 | 0.099 | 1 |
| TSH |  | 23.335 | 14 | 0.055 | 0 | NA | NA | 0.053 | -0.035 | 0.051 | 0.502 | 1 |
| TRH |  | 9.848 | 14 | 0.773 | 0 | NA | NA | 0.802 | 0.036 | 0.035 | 0.324 | 1 |
| TBG |  | -- | -- | -- | -- | -- | -- | -- | -- | -- | -- | -- |
| THRα |  | 3.739 | 10 | 0.958 | 0 | NA | NA | 0.964 | -0.016 | 0.049 | 0.756 | 1 |
| TP |  | 14.325 | 13 | 0.351 | 1 | 0.092 | 0.558 | **0.048** | 0.006 | 0.036 | 0.874 | 1 |
| TG |  | 11.736 | 9 | 0.229 | 0 | NA | NA | 0.272 | 0.062 | 0.037 | 0.130 | 1 |

*1, fixed mode effects; 2, multiplicative random effects; PSC, primary sclerosing cholangitis; TD, thyroid dysfunction; IVW, inverse-variance weighted; GD, Graves' disease; AT, autoimmune thyroiditis; TC, thyroid cancer; TSH, thyroid stimulating hormone; TRH, thyrotropin-releasing hormone, TBG, thyroxine-binding globulin; THRα, thyroid hormone receptor alpha; TP, thyroid peroxidase; TG, thyroglobulin.
